# The Health Monitoring of Bonded Composite Joints Using Both Thickness and Radial Impedance Resonance Responses

**DOI:** 10.3390/s24082508

**Published:** 2024-04-14

**Authors:** Steven P. Caldwell, Donald W. Radford

**Affiliations:** Composite Materials, Manufacture and Structures Laboratory, Colorado State University, Fort Collins, CO 80523, USA; steve.caldwell@colostate.edu

**Keywords:** structural health monitoring, single-lap-shear joint, piezoelectric sensor, composite bond, electromechanical impedance

## Abstract

With the advent of bonded composites in today’s aircraft, there is a need to verify the structural integrity of the bonded joints that comprise their structure. To produce adequate joint integrity, strict process control is required during bonding operations. The latest non-destructive joint inspection techniques cannot quantify the strength of the bond and only indicate the presence of disbonds or delaminations. Expensive and timely proof-load testing of the joints is required to demonstrate structural performance. This work focuses on experimentally evaluating joint-health monitoring with piezoelectric sensors exposed to repeated loadings until failure. Single-lap-shear composite joints are structurally tested while using sensor electromechanical impedance response as a health indicator. Based on these experiments, validation of this novel method is achieved through detailed evaluation of the sensor impedance response characteristics during loading, which enable initial and prognostic joint health assessments. The experimental results indicate that the embedded piezoelectric sensors are able to measure the sensor impedance radial and thickness resonance response changes prior to joint failure, without sacrificing the joints’ structural performance.

## 1. Introduction

As new military and commercial aircraft are being designed and fabricated, they are relying more upon the use of adhesively joined composite structures in lieu of metallic components connected with fasteners. These designs offer improvements and efficiencies ranging from manufacturing time savings, lighter weight for improved range performance, improved resistance to fatigue, and lower maintenance requirements [1,2,3]. Care must be taken during the fabrication of the bonded composite joint, as good bond integrity is only achieved by following strictly controlled processes. A lack of control (inadequate surface preparation, adherend contamination, an improper curing cycle) may produce poor structural bonds. Current joint inspection techniques are unable to detect lower-strength bonds without the presence of voids, delamination, or foreign objects [4,5,6]. The difficulty in these inspection techniques involves developing an inspection standard that will detect these weak bonds, which are sometimes referred to as “kissing” bonds and can exhibit a zero-volume disbond. Specifically, when using a structural paste adhesive to bond the composite adherends, care must be taken to not develop an amine blush formation on the adhesive surface prior to bonding. The amine component diffuses to the adhesive surface, creating a greasy, tacky layer and degrades the overall quality of the epoxy cure [7].

As the percentage of composite aircraft structures increases, so does the need for joint-health monitoring. With that, research has been more focused on producing viable techniques for assessing structural integrity [8,9,10,11,12]. These techniques address initial joint health evaluation and periodic assessment during operational usage. To date, a lot of research has been focused on detecting degradation to the external composite skin of the aircraft. Sensors are typically placed on the surface of the composite and sense damage to that surface.

Many different types of sensors may be attached to the external composite structure for monitoring. Typically, strain gages or fiber optic sensors are applied externally and used to measure abrupt load changes, which can indicate damage to the structure. Surface-mounted accelerometers and piezoelectric sensors are also used for monitoring. These sensors must have the ability to detect a local change in stiffness that can be interpreted as degradation or damage to the structure. Unfortunately, external surface-mounted sensors may not be sensitive enough to detect a change within the bond of the composite joint. And using these sensors on the outside surface of the aircraft during deployed operations leaves them prone to environmental damage, causing degraded monitoring capability. A possible monitoring option is to insert the sensor directly into the joint bond, closer to the initiation of failure. The sensor needs to be located close to the highest loaded area of the joint without any sacrifice to the joint’s pristine structural performance. 

The monitoring of piezoelectric ceramic disk sensors has been a focus of joint health evaluation [13,14,15,16]. These disks have a small footprint and are inexpensive. They serve a dual purpose in that they inject energy directly into the bonded joint and can measure the response of the joint to that energy. 

Recently, piezoelectric sensors have been inserted into bonded composite structural joints and used for health monitoring. They measure the impedance response of the joint initially after fabrication and periodically after a certain time period of loadings. A change in the joint impedance signature may indicate impending failure [17,18]. The current authors of this work performed experiments on composite single-lap-shear joints bonded with film adhesive [19]. The results of this work produced a novel health monitoring scheme that relied on an evaluation of the sensor’s through-thickness impedance resonance in addition to the standard radial response impedance measurement. The location of the sensor in the lap-shear bondline was evaluated, and it was determined that situating the sensor in the maximum-shear-stress area of the bondline (axial end points) was detrimental to joint performance compared to placing the sensor at the minimum shear stress region (center of the bond). However, a 33% degradation in failure load compared to the pristine joint was still noted with the sensor located in the center of the bond area. The cause of this strength degradation was attributed to the thickness of the sensor, which was greater than the two layers of FM300 film adhesive used to create the bonded joint. This created adhesive voids in the cured bond. Another aspect of the test coupons that affected the bond strength was the path of the thick sensor wires, protruding from either side of the lap-shear bond in the direction of the applied load. This is the area of maximum shear stress, and the wires are thicker than the sensor itself, again introducing a notable defect in the test coupon’s adhesive bondline.

The work herein focuses on a further refined set of experiments performed with sensors embedded in composite lap-shear coupons. Unlike the previous test coupons which relied on a film adhesive and elevated temperature cure, the coupons of the current study are bonded with a structural paste adhesive, enabling room-temperature curing and negating the need for vacuum bagging. Additional set experiments are performed in an attempt to address the deficiencies encountered in the previous coupons which led to observed bond strength reduction, which was mainly attributed to the void-rich bond due to the sensor thickness, and the thick wires being located through the maximum-shear-stress region. This work is performed to show that the embedded sensor does not degrade the bond strength and to further elaborate upon and address the structural health monitoring capability of using the electromechanical impedance primary resonance response characteristics (both radial and thickness) during successive loadings. A novel approach of calculating impedance resonance modal damping factors and peak amplitude trends with the incremental test loads is evaluated. 

## 2. Experimentation/Results

These experiments are performed to evaluate the capability of the composite single-lap-shear-joint embedded sensor impedance responses enabling refinement of the bond health assessment methodology. It is of note that use of a single-lap-shear joint is the most common load test for sensor performance evaluation. The experiments are also performed with pristine joints to allow for the evaluation of any joint performance degradation due to the in situ sensor. 

### 2.1. Free Sensors’ Electromechanical Impedance Responses

The piezoelectric sensor disks used in these experiments were ordered from APC International (Mackeyville, PA, USA) and are solid disks consisting of a highly purified lead zirconate (PZT) ceramic [20]. These were the same model of sensors that were used in the previous set of experiments with the film adhesive bonds. The only difference was in the soldering of the lead wires that connect to the spectrum analyzer. To apply the voltage to the sensor disk, one lead was soldered to one surface (positive) and one lead was soldered to the other surface (negative). In the previous experiments, the leads were soldered at the same location on each surface and run off together from one side of the disk. The leads were thick (0.50 mm) and possibly added to the bondline defect in the last experiments. This time, APC was required to bond them on the opposite side of the surface so that one lead extended off one side and the second lead off the other side. This minimized the thickness footprint of the leads in the bond. 

**Figure 1:**
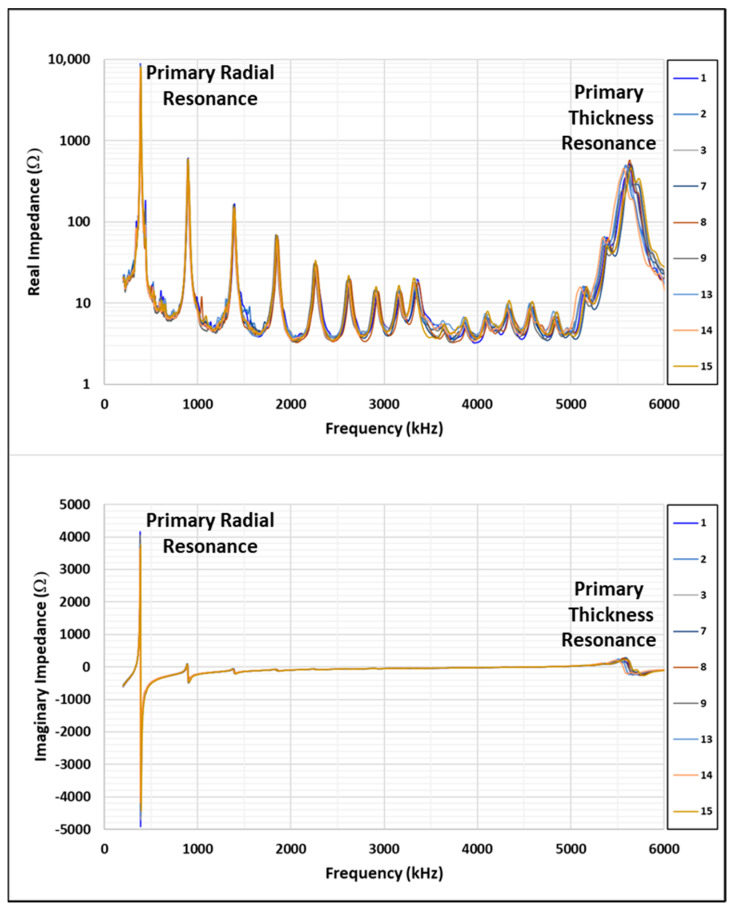
Free sensors’ impedance responses.

A total of 15 sensors were obtained from APC international. The electromechanical impedance for all sensors was measured while the sensors were supported in the soft packing material (essentially a free-boundary state). The sensor impedance response is fairly consistent across all sensors when comparing the primary radial and thickness resonance frequency and amplitude. Based on the response consistency, 10 of the sensors were selected for bonding and the others were set aside as spares. The free sensors’ impedance responses are shown in Table 1 and a frequency response plot is shown in Figure 1.

### 2.2. Test Coupon Fabrication

**Figure 2:**
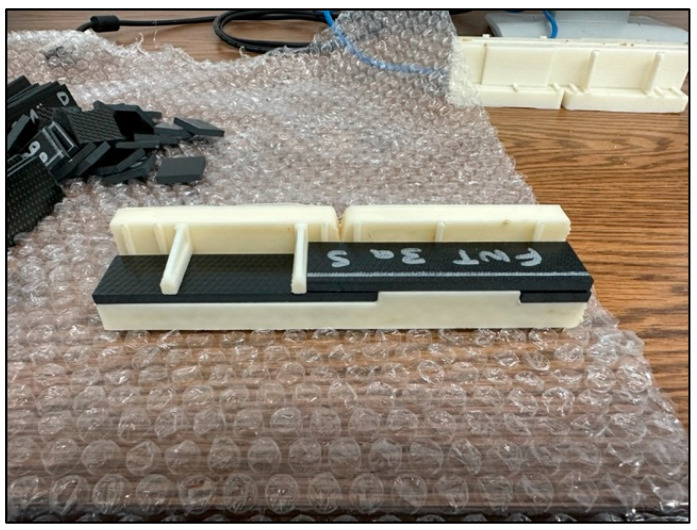
Single-lap-shear adherends fit into bond fixture.

The single-lap-shear coupons were fabricated from composite adherends that consist of 12 plies of T650-35/5320-1 carbon/epoxy 8HS woven fabric prepreg in a quasi-isotropic layup ((45/0)_3_)_S_. The adherends used the Solvay CYCOM 5320-1 toughened epoxy resin system and were cured in a vacuum bag at 177 °C to form a panel [21]. The panel was then cut into test coupon adherends with a diamond saw. The test adherends were bonded together using LOCTITE EA 9394 AERO epoxy paste adhesive [22]. EA 9394 is a two-part structural paste adhesive which cures at room temperature and does not require vacuum bagging. The room-temperature curing takes 3–5 days at 25 °C to achieve nominal mechanical performance. It is acceptable to accelerate the curing by placing the coupons in an oven at 66 °C for 1 h. A total of 12 single-lap-shear test coupons were fabricated (eight pristine and four with bondline-embedded sensors). The sensors were located in the center of the shear bond with the lead wires running out of the coupon perpendicular to the load axis. This was done to minimize any degradation effect on the structural performance by locating the sensor/wires in the area of minimum shear stress. The single-lap-shear adherends were placed into a 3D-printed fixture for assembly and curing. The bond fixture was designed to maintain the coupon geometry and control the bondline thickness. The target bondline thickness was 0.71 mm, with the fixture designed to give the same bondline thickness between the pristine and embedded sensor coupons. Figure 2 shows the single-lap-shear adherends fit into the 3D bond fixture.

**Figure 3:**
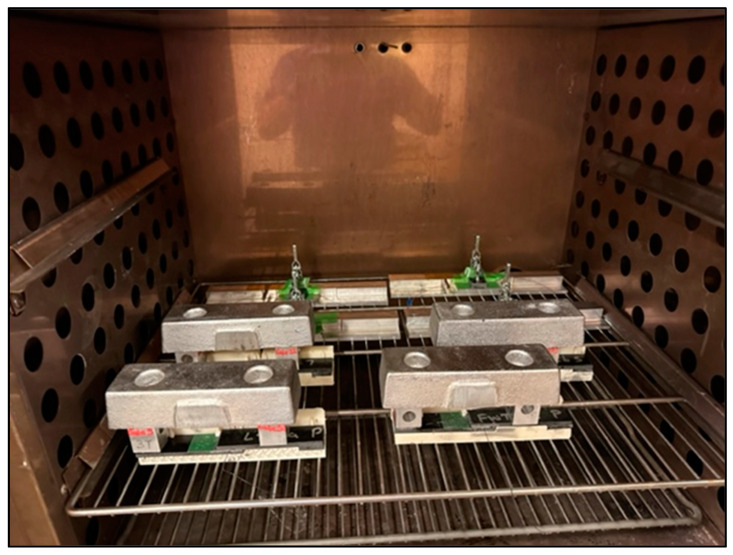
Coupons placed in the oven for accelerated curing.

The two-part structural paste adhesive was mixed together, applied to the prepped composite adherend surfaces and fit into the bond fixture. The coupons were placed in the oven for accelerated curing at 66 °C for 1 h, as shown in Figure 3. There were four lap-shear bond fixtures; therefore, three cures were required to fabricate 12 test coupons. Weights are placed on the coupons to ensure consolidation of the adhesive.

**Figure 4:**
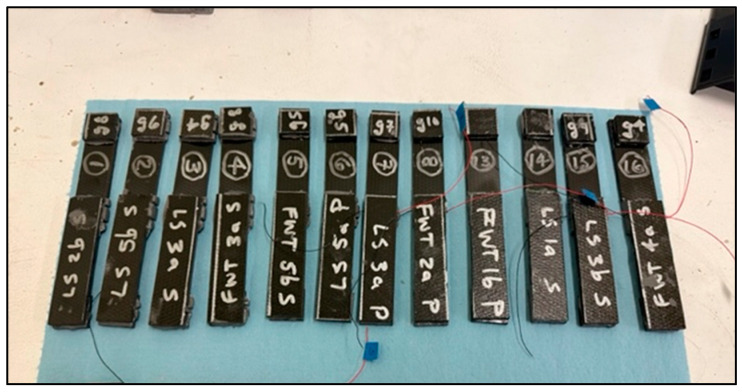
Cured test coupons.

The cured test coupons are shown in Figure 4. The sensor lead wires can be seen coming out of the bonded coupons with embedded sensors (positive—red and negative—black).

**Figure 5:**
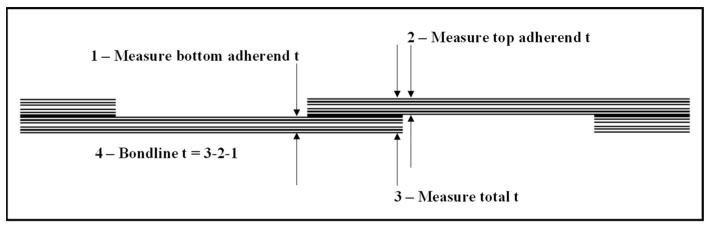
Sample coupon bondline thickness measurement process.

The bondline thickness for the three batches of lap-shear coupons was measured and evaluated for consistency and proximity to the target of 0.71 mm. Figure 5 shows the geometry of the sample and the thickness measurement process, in which a micrometer was used to measure the thickness at the indicated locations; then, the sample bond thickness was calculated. The first batch of coupons cured were all pristine (no sensor) with an average bondline thickness of 0.74 mm, which was close to the target. For batches 2 and 3, the average bondline thickness was 0.58 mm for the pristine coupons and 0.69 mm for those with embedded sensors. 

### 2.3. Pristine Single-Lap-Shear Coupon Experiments

**Figure 6:**
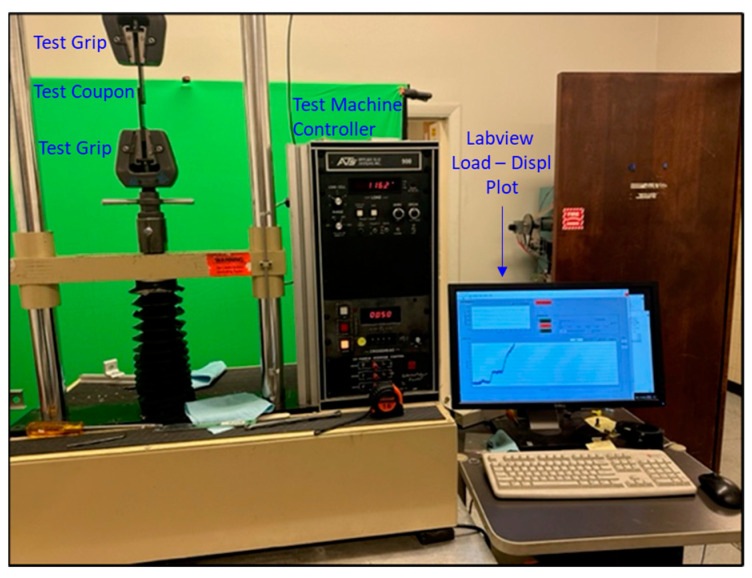
Pristine lap-shear coupon loaded in the test machine.

The pristine single-lap-shear coupons were tested mechanically in the ATS 900 universal test machine shown in Figure 6 using the ASTM D5868 FRP single-lap-shear test procedure [23].

**Figure 7:**
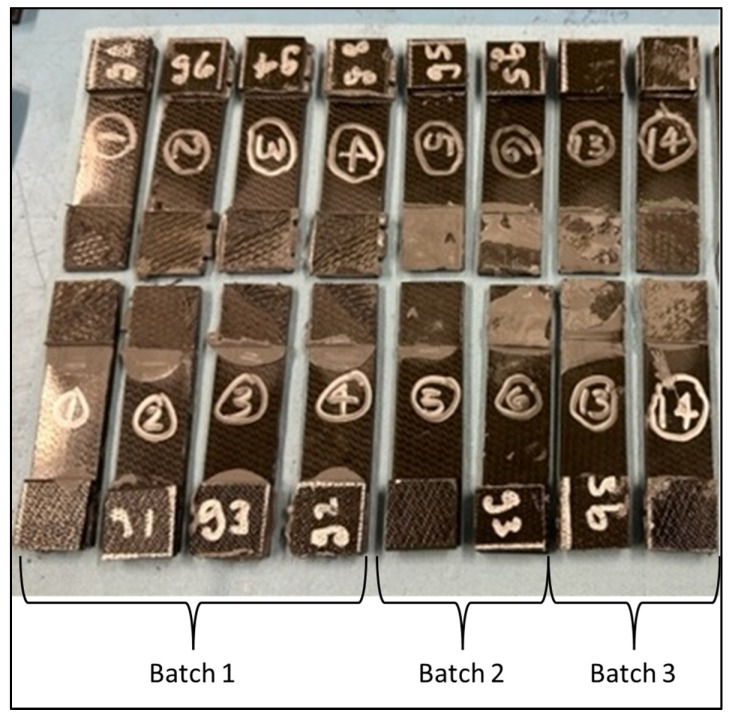
Failed pristine lap-shear coupons.

**Figure 8:**
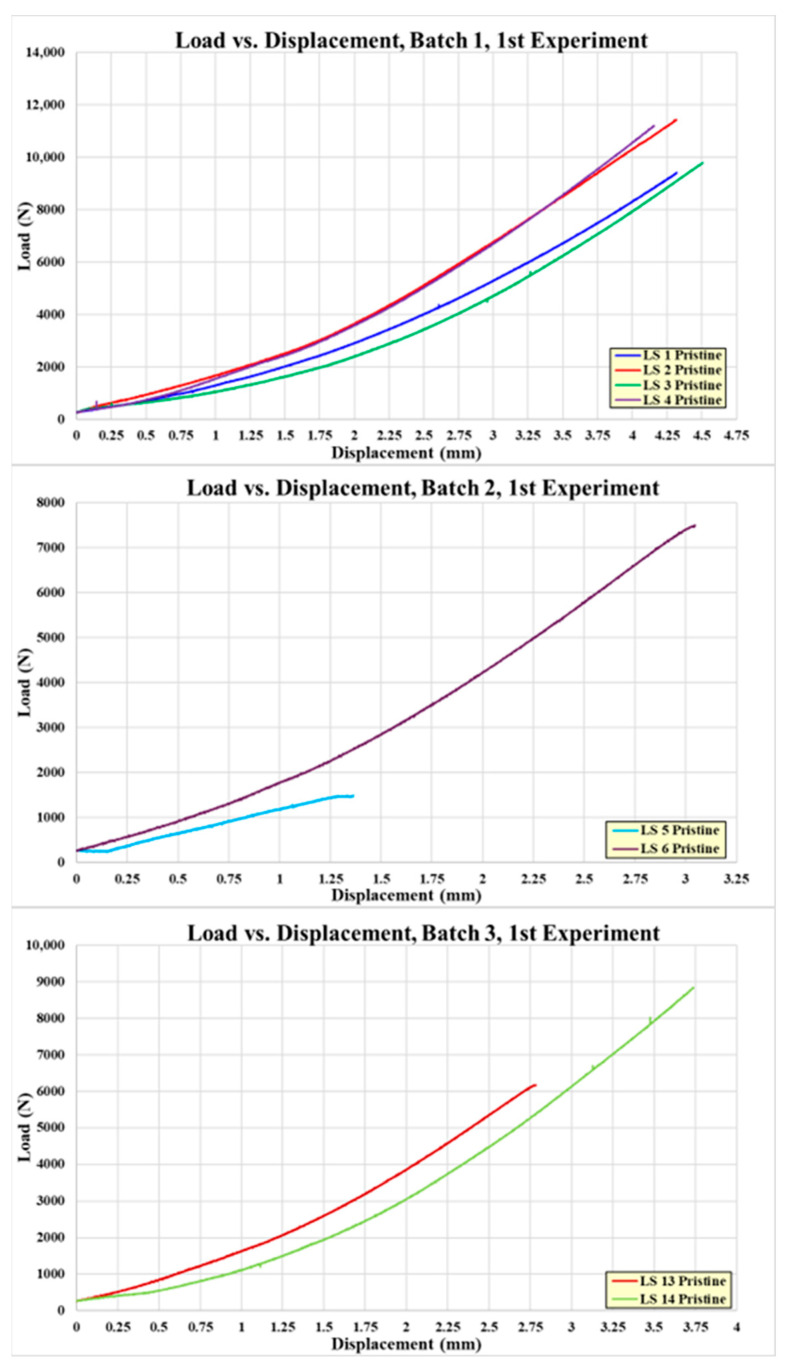
Load-versus displacement curves for pristine coupons.

There were a total of six pristine lap-shear coupons tested, four from the batch 1 adhesive, two from the batch 2 adhesive, and two from the batch 3 adhesive. Table 2 shows the test failure load for each pristine coupon. The average failure load for the batch 1 coupons is 10,806 N, batch 2 is 4613 N, and batch 3 is 7543 N. The failed pristine coupons are shown in Figure 7. Note that each adherend is oriented so both sides of the failure surface may be observed. The batch 1 coupons exhibit higher failure loads and indicate a degree of composite adherend failure, corresponding to bonds with good structural integrity. The batch 2 and 3 coupons failed at considerably lower loads than the batch 1 coupons. This is attributed to inadequate curing of the paste adhesive, which most likely occurred due to incomplete mixing of the two parts of EA 9394 before bonding. In Figure 7, the batch 2 and 3 failures appear to be a mixture of adhesive and cohesive and a combination of both modes. The failed coupon adhesive has a rubbery feel, which lends credence to the idea of incomplete adhesive curing. Figure 8 shows the load-versus-displacement curves for the eight coupons up to failure aligned with the curing of each batch.

### 2.4. Embedded Sensor Single-Lap-Shear Coupon Experiments

**Figure 9:**
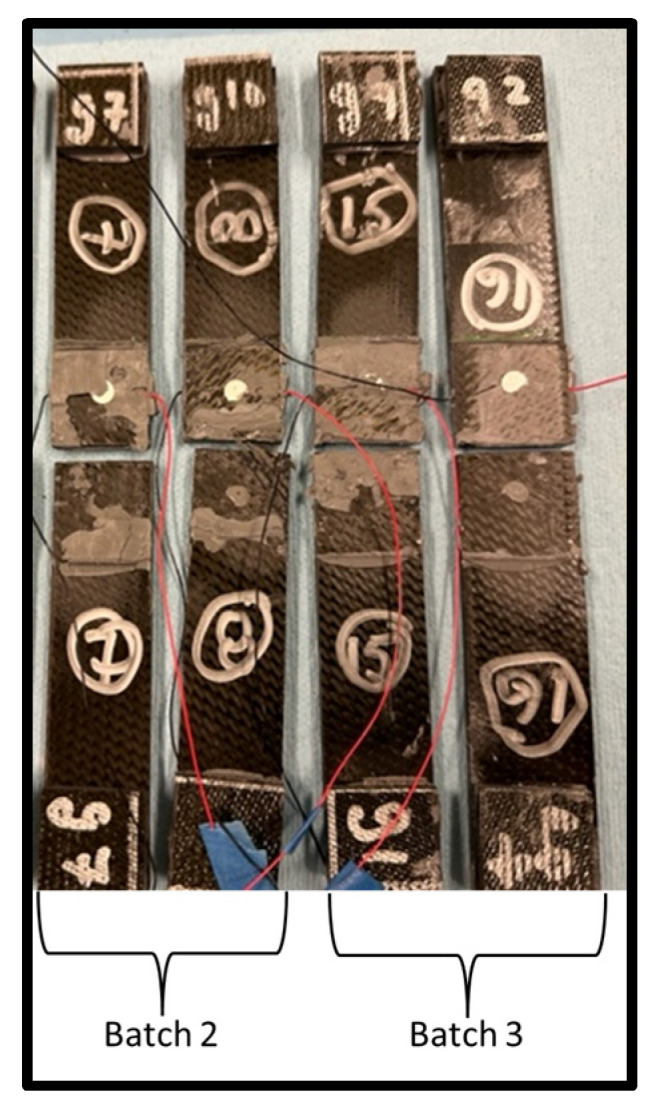
Failed embedded sensor lap-shear coupons.

**Figure 10:**
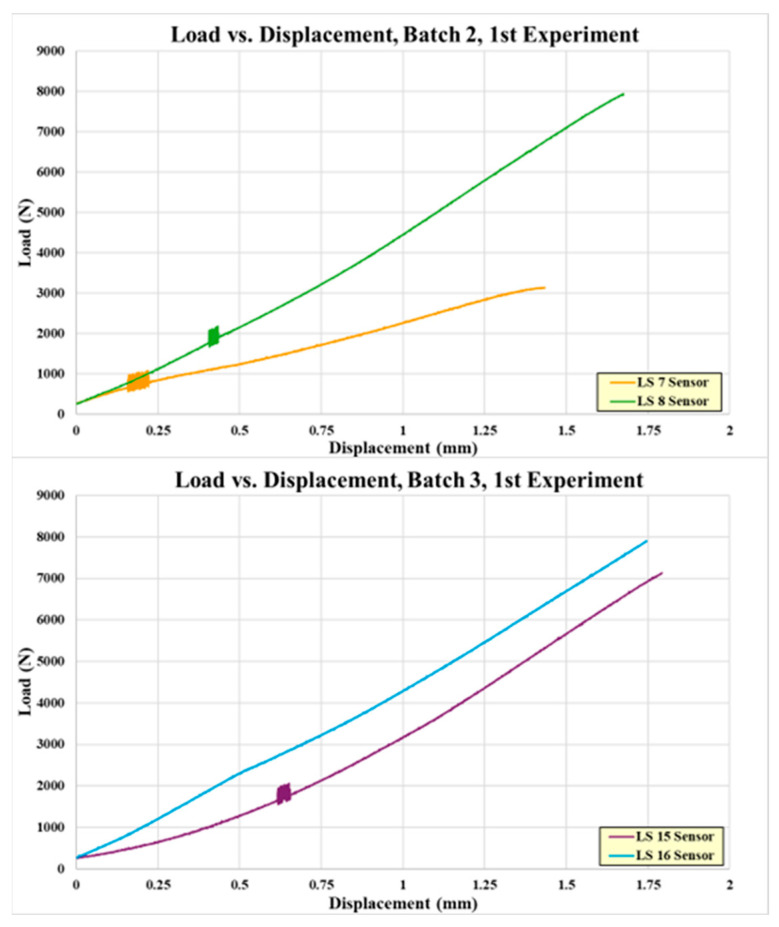
Load-versus-displacement curves for embedded sensor coupons from 1st experiments.

There were four coupons fabricated with embedded sensors, two in the batch 2 curing and two in the batch 3 curing. The coupons were tensile tested incrementally until failure, and between loads, the electromechanical impedance was tested and recorded. Table 3 shows the embedded sensor test coupon failure loads. Coupon #7 exhibited a low failure load, indicating a structurally weak bond similar to pristine coupon #5. Figure 9 shows the failed embedded sensor coupons. Their failure appears to be purely an adhesive failure, supporting the idea of inadequate adhesive mixing and curing. The rest of the embedded sensor coupons exhibited higher failure loads, fairly consistently. Figure 10 shows the load-versus-displacement curves for the four embedded sensor coupons up to failure.

### 2.5. Embedded Sensor Electromechanical Impedance Tests

**Figure 11:**
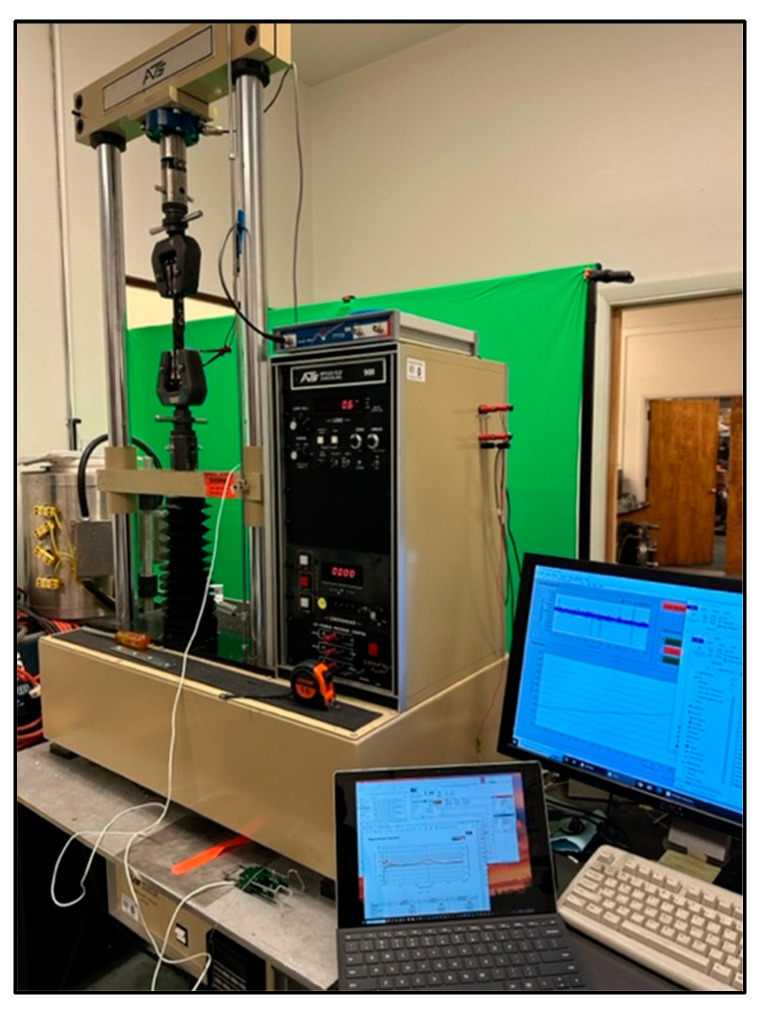
Fixture with installed embedded sensor coupon connected to analyzer.

The embedded sensor coupons were each installed in the test fixture and the baseline embedded sensor impedance was measured. They were loaded initially to 2222 N, and then, unloaded, and the impedance was measured with the coupon installed in the test fixture. The load was increased incrementally by 445 N, and then, unloaded, and the impedance measured, and this was repeated until failure. Figure 11 shows one of the coupons installed in the test fixture with the sensor lead wires connected to the analyzer and a laptop.

**Figure 12:**
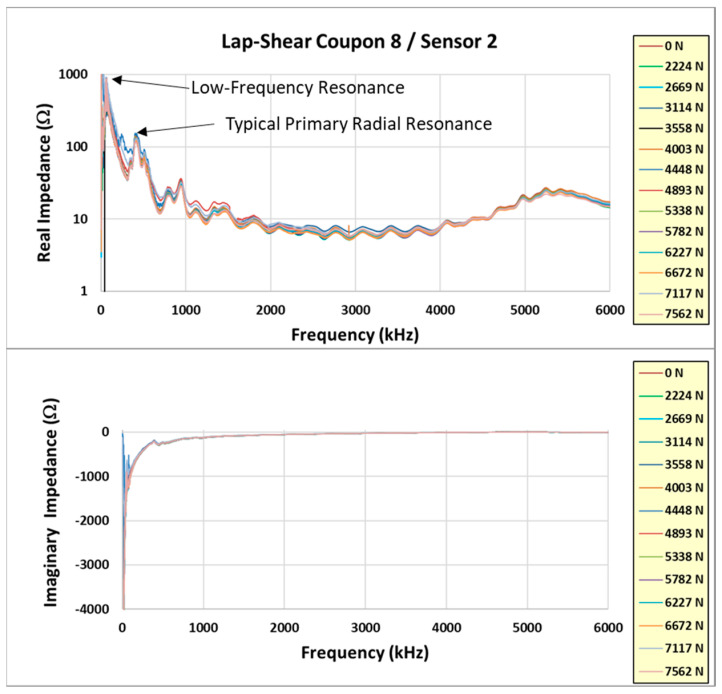
Coupon #8 electromechanical impedance response.

Normally, the electromechanical impedance primary radial resonance increases due to the constraint of the sensor in the lap-shear joint (as opposed to a free state). According to previous test experience with film adhesive bonds, this resonance is >400 kHz. There was a much lower primary resonance measured in these experiments. This is shown in Figure 12 for coupon #8. The other embedded sensor coupons exhibited similar results.

**Figure 13:**
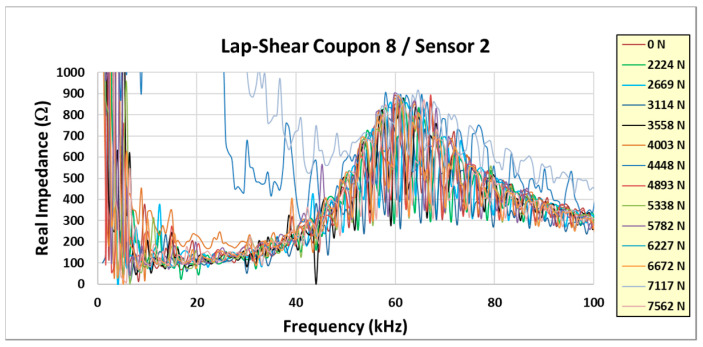
Closeup of low-frequency response.

It is noted in Figure 12 that there is a lower-frequency response that seems to dominate the response and overshadow the normal 400 kHz resonance. Figure 13 shows a closer look at the real part of the impedance response up to 100 kHz. The response is concentrated around 60 kHz and contains a high-frequency large-amplitude oscillation. This phenomenon is attributed to interference from the test fixture and was not noticed until the post-test data review. Unfortunately, the presence of this phenomenon negatively affected the ability of the embedded sensor to monitor the structural health of the lap-shear bond. The typical damage index calculation was not meaningful and did not predict the failure of the tested coupons. Due to this, it was decided to fabricate more coupons and repeat the tests.

### 2.6. Second Round of Lap-Shear Coupon Fabrication

**Figure 14:**
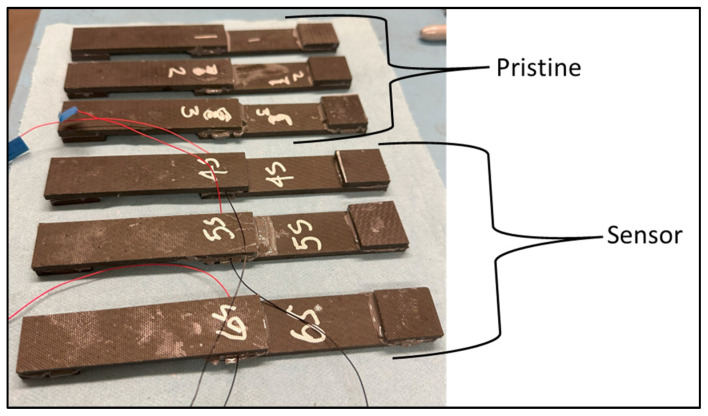
Second set of fabricated coupons.

Based on the results of the initial experiments, it was decided to fabricate a second set of paste-adhesive-bonded single-lap-shear coupons and repeat the tests. Three pristine coupons and three with embedded sensors were fabricated using the 3D-printed bond fixtures and EA 9394 paste adhesive. Figure 14 shows the completed test coupons.

The sensors were all located at the center of the lap-shear bond, similar to the previous coupons, and the lead wires exited the bond area at the lowest shear stress (perpendicular to the load direction). The average bondline thickness for the pristine coupons was 0.74 mm, and it was 0.83 mm for the embedded sensor coupons. The bondline thickness between the pristine and sensor coupons was closer than in the previous set of experiments (batch 2 and batch 3 cures). Note the 6% bondline thickness increase with the addition of the sensor compared to the 17% thickness increase in the prior paste adhesive experiments. 

### 2.7. Second Set of Single-Lap-Shear Coupon Experiments

**Figure 15:**
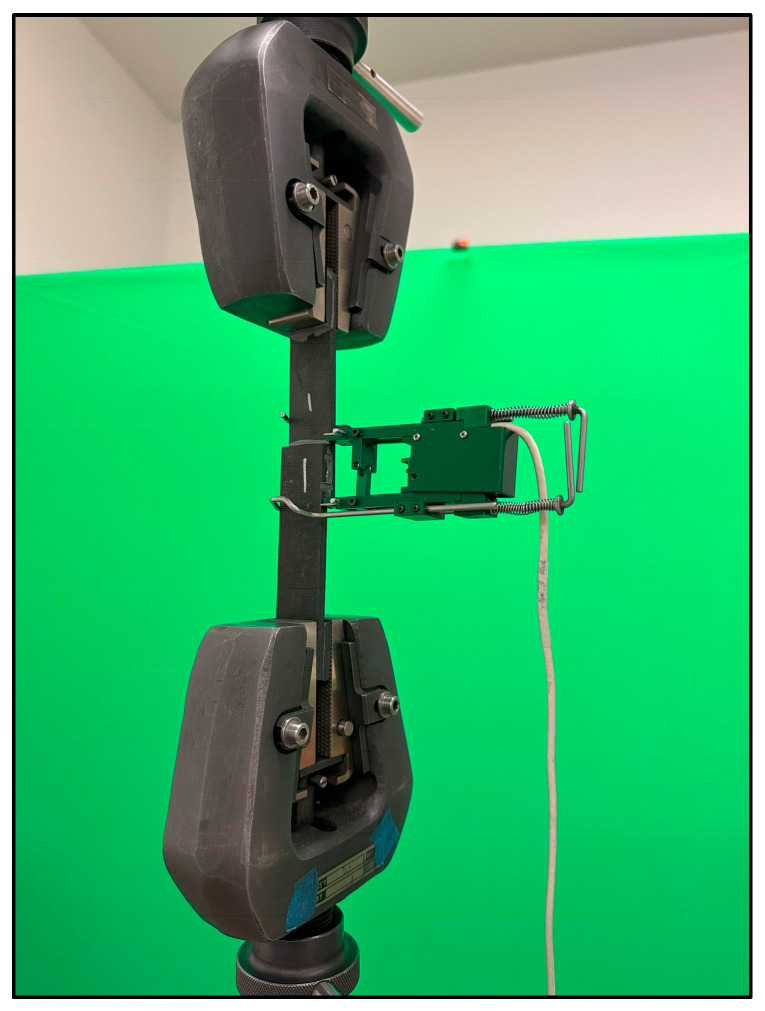
Coupon #1 loaded in the test machine.

The pristine coupons were each inserted into the test machine and loaded until failure, as shown in Figure 15 for coupon #1. The average failure load for the pristine coupons is 8201 N. The embedded sensor coupons were tested incrementally, similar to the first set of experiments, until failure. The average failure load for the embedded sensor coupons is 8289 N. Note that the electromechanical impedance was measured between the incremental load applications while the coupon was in the unloaded state. Table 4 shows the failure load for each coupon test.

**Figure 16:**
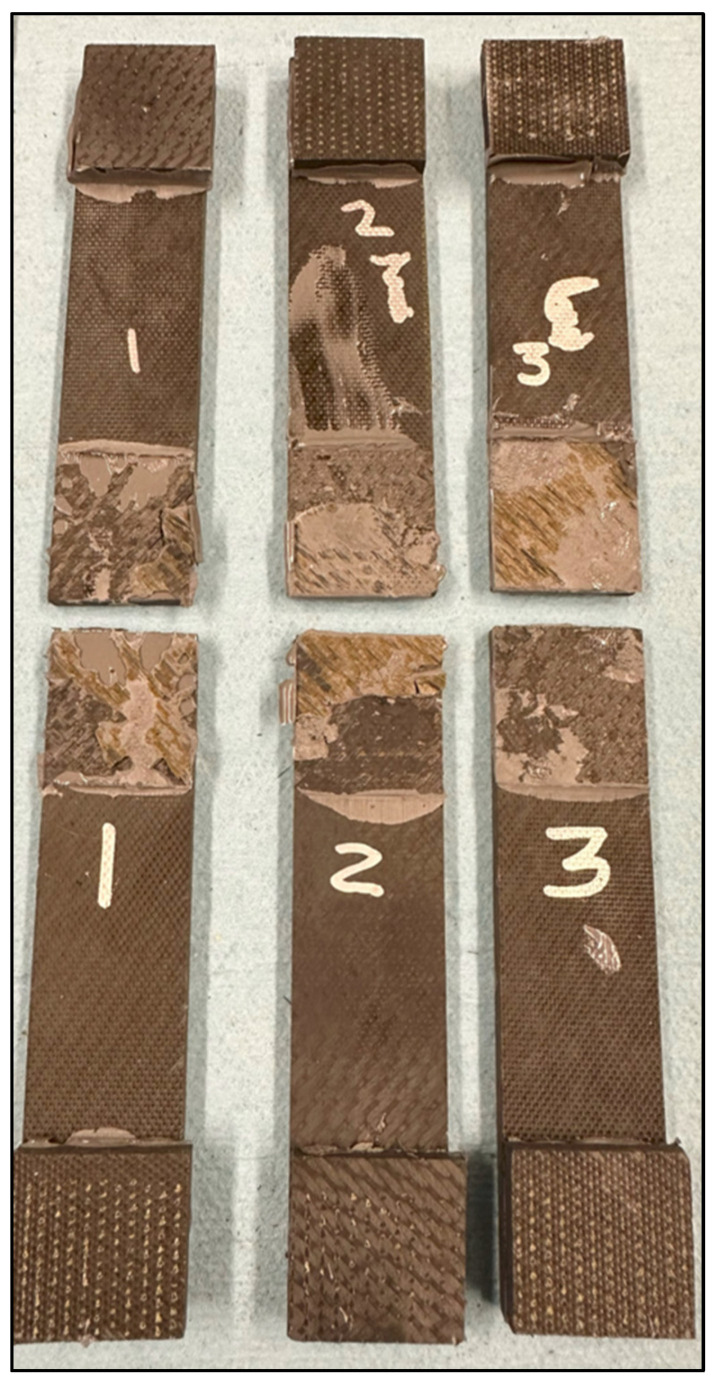
Failed pristine coupon adherends.

**Figure 17:**
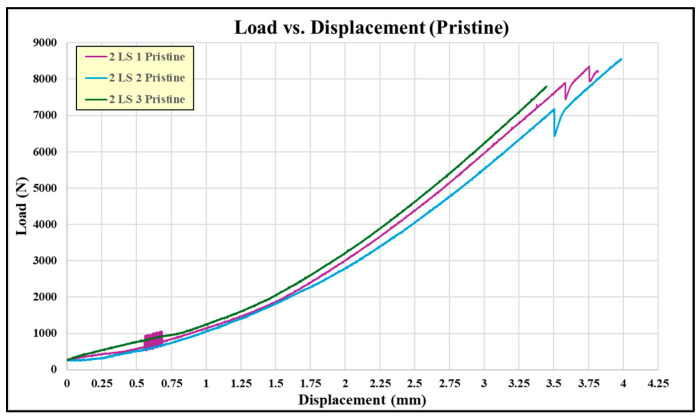
Load-versus-displacement curves.

The failure loads between the pristine and embedded sensor coupons are much closer than the loads in the first set of paste bond experiments, with only a 1% difference in the average failure load. Figure 16 shows the failed pristine coupons, which all look similar, exhibiting mixed-mode failure (adhesion/cohesion/fiber tear), and Figure 17 shows the load-versus-displacement test curves.

**Figure 18:**
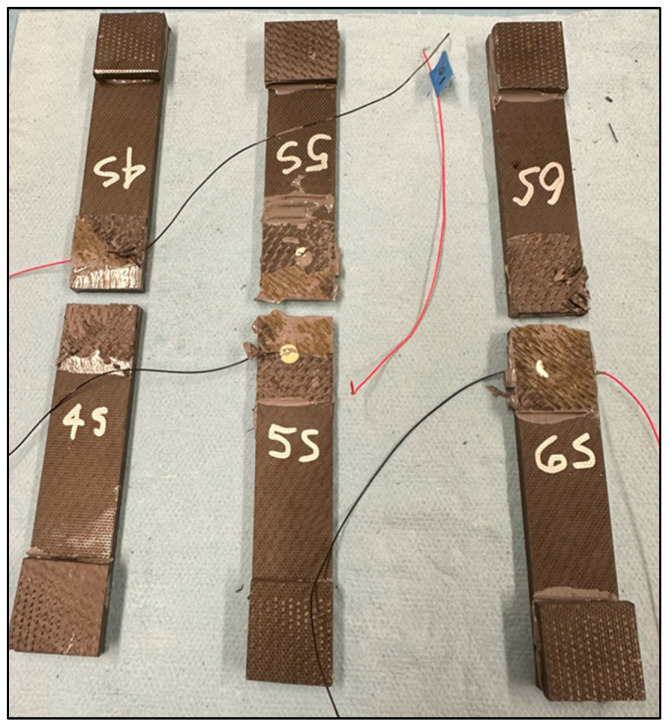
Failed sensor coupon adherends.

**Figure 19:**
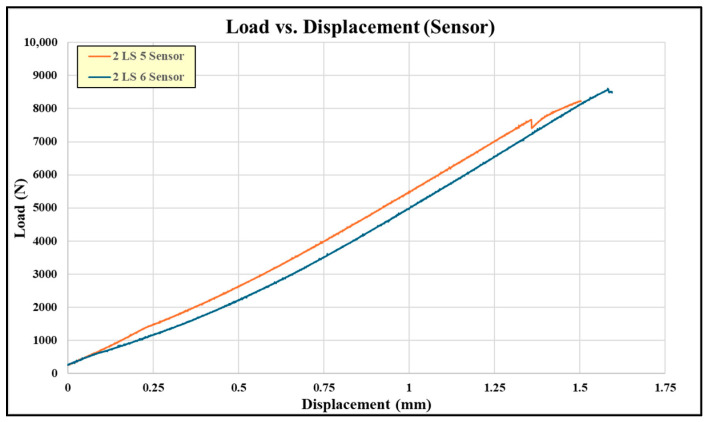
Load-versus-displacement curves for embedded sensor coupons from 2nd experiments.

Figure 18 shows the failed embedded sensor coupons, which exhibit failures similar to the pristine coupons. Note that the failure surfaces include a degree of composite adherend failure which is normally considered to be a “good” structural bond. The load-versus-displacement curves for these coupons are shown in Figure 19. Note that there is not a curve for coupon #4 due to the test load being continuously held during impedance measurement.

**Figure 20:**
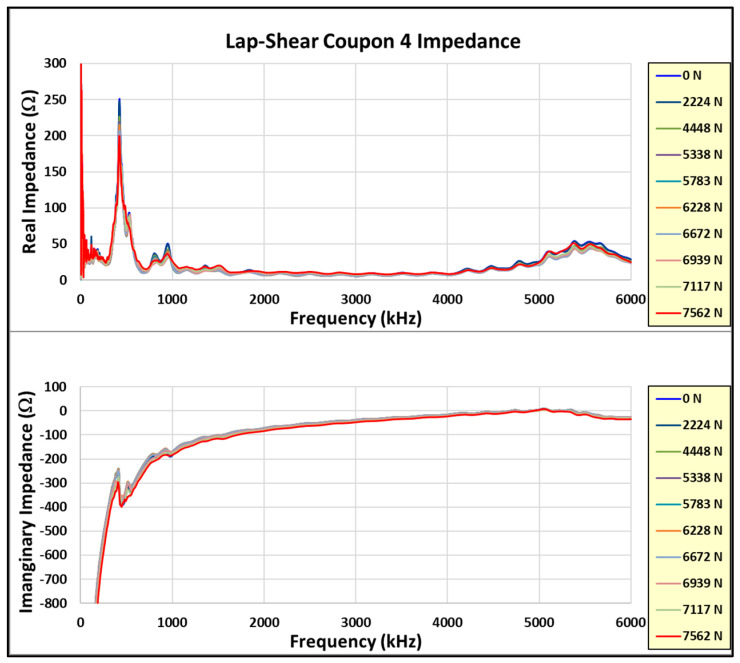
Coupon #4 sensor impedance/load results.

**Figure 21:**
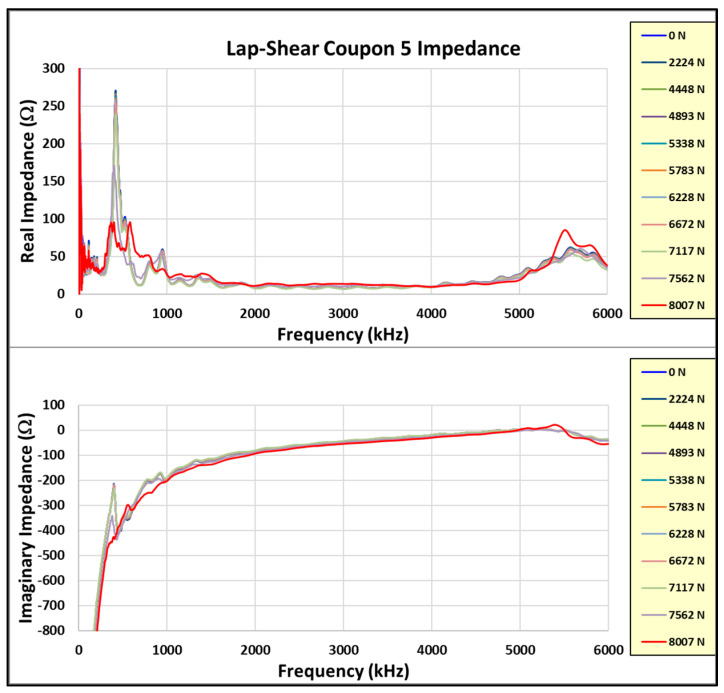
Coupon #5 sensor impedance/load results.

**Figure 22:**
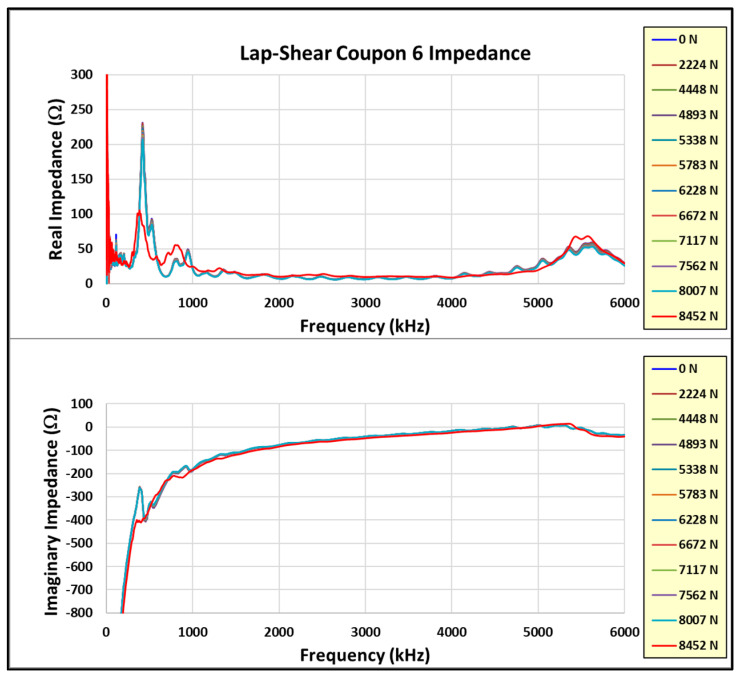
Coupon #6 sensor impedance/load results.

Figure 20, Figure 21 and Figure 22 show the incremental impedance measurements (real and imaginary) for each of the three series of embedded sensor coupon tests, with a curve for each impedance measurement. Note that the impedance is measured after the coupon is unloaded.

Similar patterns are observed in the primary radial and thickness impedance responses as the joint load increases for coupons #5 and #6, but not coupon #4. Coupon #4 was not unloaded during impedance measurements, whereas coupons #5 and #6 were measured in the unloaded state. Therefore, the impedance must always be measured in the unloaded state.

## 3. Discussion

The focus of this experimental work is to assess the viability of embedded piezoelectric sensors within the bondline of single-lap-shear composite joints to monitor the structural health of the bond and anticipate impending joint failure from repeated loadings. Previous experiments were performed on embedded sensors in single-lap-shear composite joints with film adhesive bonds, showing the promise of health monitoring using radial and thickness impedance response characteristics during loading [19]. However, the structural performance of these joints was degraded due to the inclusion of the sensor in the bond. To further evaluate this performance, a second set of experiments were performed using a structural paste adhesive with a thicker bondline to maintain the structural performance of the joint with the embedded sensor. The use of the paste adhesive with a 3D-printed bond fixture acted to control the bondline thickness and resulted in similar, uniformly thick bondlines between the pristine and embedded sensor bonds. The single-lap-shear failure loads experienced with these sensor coupons were within 1% of the pristine coupons and exhibited similar failure modes. The correction from the previous experiments aimed to create a bondline that was double the sensor thickness, thus allowing the adhesive to fully incapsulate the sensor and reduce the adhesive voids experienced in the prior film adhesive bonds. Also, the sensor lead wires were routed out of the bond in the area of minimum shear stress and were not routed out in a single bundle. They were routed out one wire per side of the coupon. This helped to reduce the wire footprint on the bonding surfaces. In order to expand on the in situ sensor’s ability to monitor bond health during repeated loadings, the primary impedance resonance for radial and thickness responses was evaluated to determine damping and amplitude trends and variations. 

Three embedded sensor lap-shear coupons were tested to failure using incremental increasing load applications. The impedance was measured between loads, normally in the unloaded state but still installed in the test fixture. Coupon #4 was tested first with the measured electromechanical impedance installed in the test fixture but unloaded. The coupon survived nine successive load increments and failed at 8326 N. For this coupon, it was decided to measure the impedance of the sensor while the coupon was loaded (pause in the upload). This was done to save time, but unfortunately, this corrupted the impedance measurements and these data were not used for damage index evaluation.

**Figure 23:**
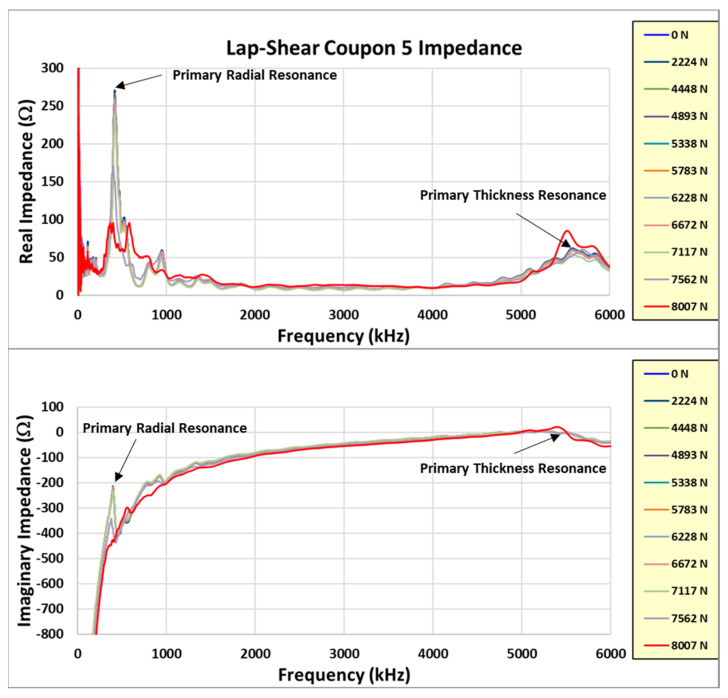
Coupon #5 impedance measurements.

Learning from the experience with coupon #4, coupons #5 and #6 were incrementally loaded with the impedance measured between loads in the unloaded state. The test data from these two coupons are reviewed in more detail. Reviewing the coupon #5 results first, Figure 23 shows the real and imaginary impedance measurements up to failure. Note the appreciable resonance shift for the primary resonances (radial and thickness) on the highest recorded load impedance.

**Figure 24:**
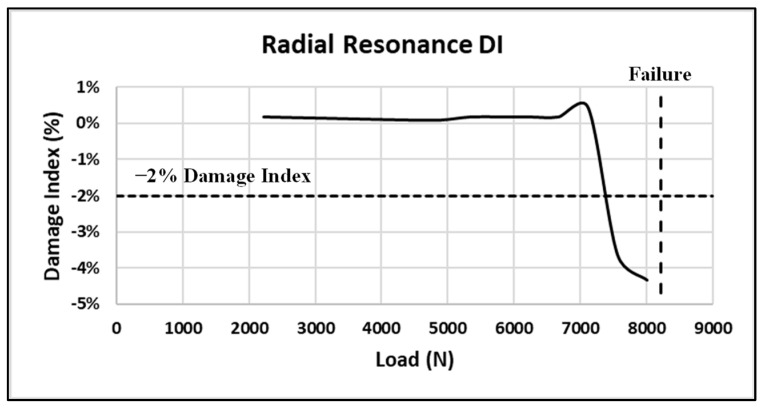
Coupon #5 radial-mode DI.

The initial method used to evaluate the structural health of the bond was the damage index (DI), which is the root-mean-square deviation (RMSD) of the real part of the measured impedance resonance as the load is increased [13]. The equation is given as follows:

(1) $$RMSD=\sqrt{\frac{\sum_{i=1}^{n}{\left[Re\left(Z_{n}\left(\omega_{i}\right)\right)-Re(Z_{u}\left(\omega_{i}\right))\right]}^{2}}{\sum_{i=1}^{n}{\left[Re(Z_{n}\left(\omega_{i}\right))\right]}^{2}}}$$

where *Z_n_* is the initial healthy bond impedance and *Z_u_* is the measured bond impedance after each load is applied. The damage index is normally evaluated only for primary radial impedance resonance but may be evaluated for any impedance resonance. Typically, a DI of −2% or greater indicates impending coupon failure. For these experiments, the primary radial and thickness impedance modes were evaluated. Examining the sensor radial resonance first, Figure 24 shows the primary radial-mode DI as the load approaches failure (note that the failure load is 8221 N, designated as a dashed vertical line in the figure).

**Figure 25:**
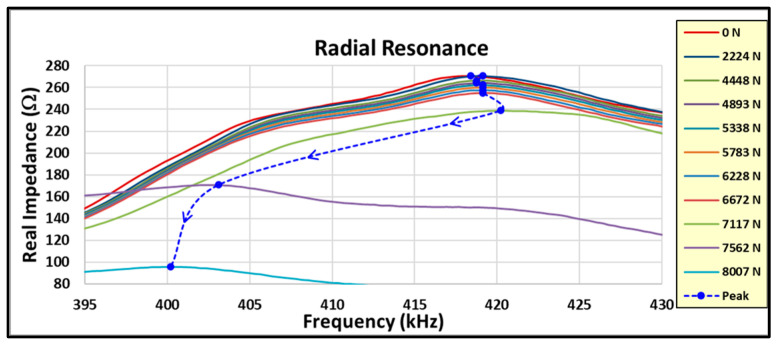
Radial resonance response between loads.

Note initial increase followed by a rapid change (decrease) in the DI prior to failure. The initial increase in the primary resonance may indicate a joint consolidation prior to the onset of degradation. The horizontal dashed line at a DI of −2% is the normal estimated threshold used to indicate impending failure. This coupon achieved a DI of −4.3% prior to failing. Examining the radial resonance response more closely between loadings to include the amplitude and not just the frequency change produces Figure 25 for the coupon #5 data.

**Figure 26:**
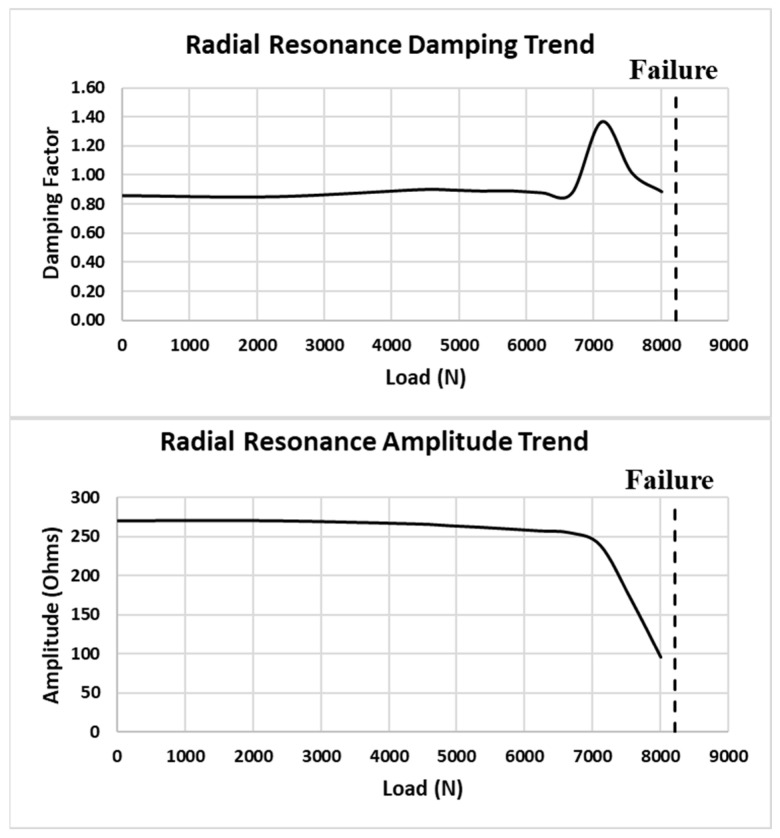
Coupon #5 radial resonance damping and amplitude trends.

The dashed line connects the peak amplitude of the radial resonance for each load, with the arrows designating the increasing load direction. Note that the amplitude decreases and the modal damping increases for each incremental load and as the frequency change increases. The damping trend is estimated by using an approach similar to the half power method for modal damping calculation. Figure 26 shows an estimate of the modal damping trend and amplitude of the radial resonance as the load level increases to failure.

There is a large decrease in the modal amplitude as the coupon approaches failure. This is similar to the DI as the coupon passes the 7000 N load value. It is of note that an increase in damping is observed at a lower load level (<7000 N). This adds valuable insight to the health monitoring ability of the impedance response, possibly suggesting impending failure earlier than the DI evaluation indicates. 

**Figure 27:**
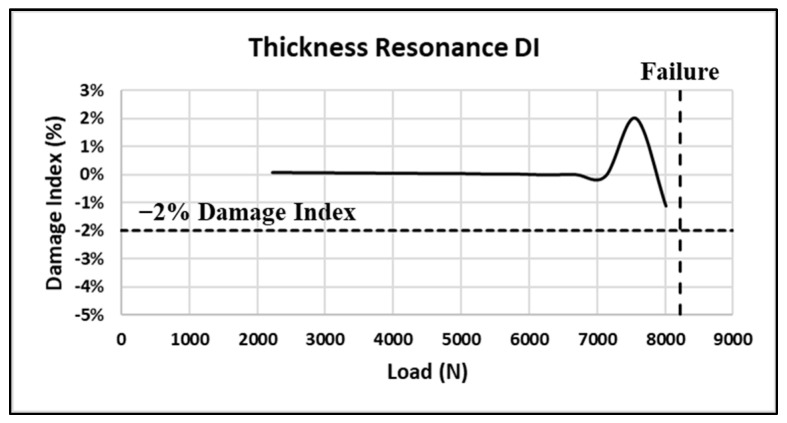
Coupon #5 thickness resonance DI.

**Figure 28:**
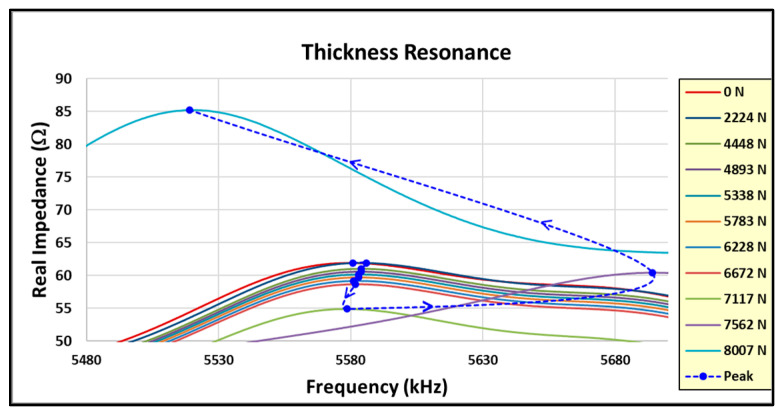
Coupon #5 thickness resonance response between loads.

In a novel manner, this same approach is applied to the primary thickness impedance resonance. Figure 27 shows the standard DI for the thickness resonance. Note that the DI increases to +2% before decreasing on the final load. Figure 28 shows a closeup of the thickness resonance during loading. The initial uptick in response is more prominent than the radial mode exhibited, indicating hypersensitivity in the thickness direction, which could mainly be attributed to the higher resonance frequency.

**Figure 29:**
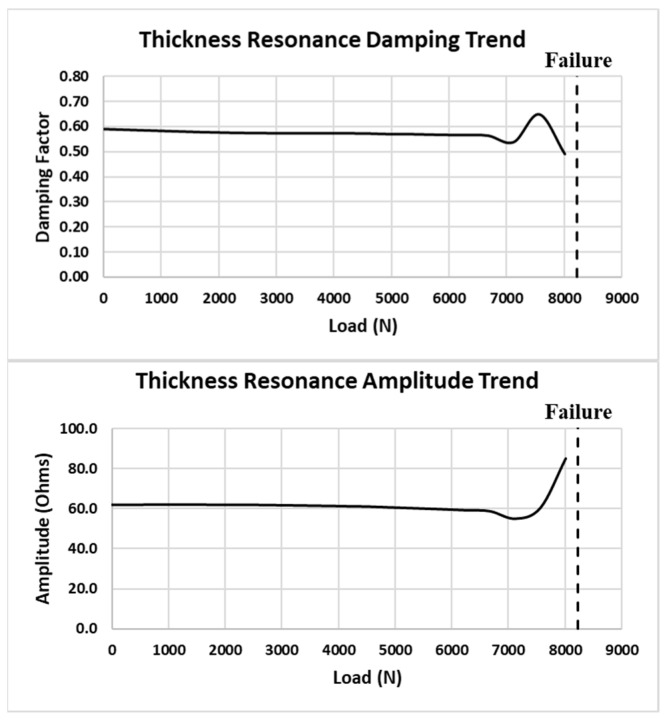
Coupon #5 thickness mode damping and amplitude trends.

In Figure 28, note the initial decrease in amplitude at a relatively constant frequency followed by an abrupt amplitude increase. This is a different response than what is observed in the radial resonance and indicates that the joint becomes more responsive in the thickness direction, which may be due to the higher shear stress as the sample approaches failure. The resonance damping and amplitude trends for the loads are shown in Figure 29. 

**Figure 30:**
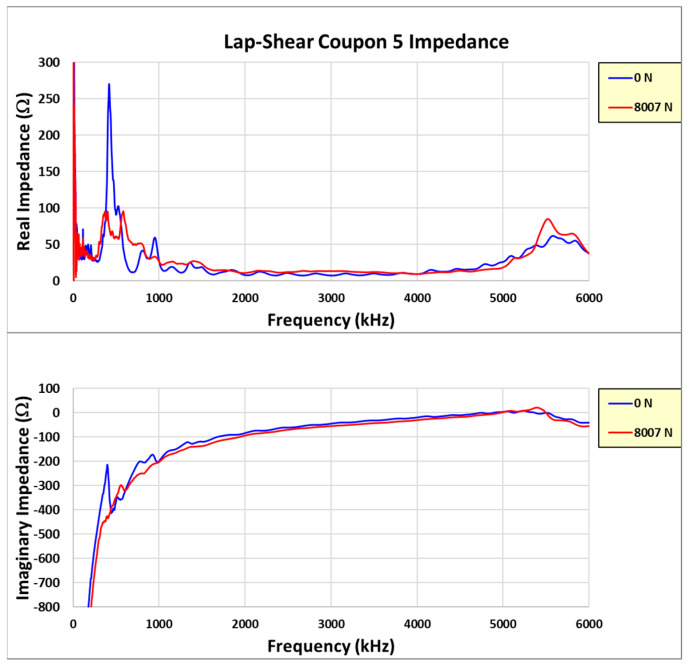
Coupon #5 initial and final impedance measurements.

Another way to view the change in impedance is to only plot the initial and final load measurements. This shows the change clearly without the incremental load plots included, as seen in Figure 30. The creation of two radial resonances from the initial radial mode is observed in the plot. The final load’s radial impedance responses are much lower in amplitude than those of the initial load (both real and imaginary), which is primarily attributed to the increase in damping as the joint load increases prior to failure. The final load shows that the thickness mode is clearly dominant and more decoupled from the radial-mode harmonics, which appear to have dissipated since the lower-amplitude modes were created.

A similar data analysis was performed on the coupon #6 test data, which concludes with similar results. Thus, based on these experiments, validation of our novel method is achieved through detailed evaluation of the sensor impedance response characteristics during loading, confirming that it provides initial and prognostic joint health assessments.

## 4. Conclusions

The experiments conducted in this study successfully proved the prognostic health monitoring capability of embedding piezoelectric sensors at the center of the bond in single-lap-shear composite joints. The structural performance of the joint was not degraded due to the addition of the sensor as it was in the prior set of film-adhesive-bonded experiments. This was due to an increase in adhesive in the bond to fully incapsulate the sensor and decrease the abundance of voids experienced in the prior experiments. Paste adhesive was used for coupon bonding in lieu of film adhesive to more efficiently fabricate the coupons (no vacuum bagging). Another key improvement to the performance of the embedded sensor coupon was running the sensor wires out of each side of the bond in the minimum range of the joint shear stress. The first set of paste adhesive coupons experiments were not able to produce meaningful impedance data for evaluation due to signal interference from the test fixture to the coupon sensor. This fixture interference was cleared for the second set, allowing for a thorough data review and the use of the experiments to validate the method through detailed evaluation of the sensor impedance response characteristics. Evaluating the higher-frequency thickness mode proved to help improve the joint monitoring capability by providing additional information; when combined with radial resonance evaluation, it gives a complete picture of joint health status. Adding the additional quantities of damping and amplitude trends provide more indicators of bond health, as summarized in the discussion, and may provide an earlier warning of impending joint failure than solely using the DI as an indicator.

## Figures and Tables

**Table 1:** Free sensors’ measured impedance resonances.

Sensor	Radial (kHz)	Amplitude (kOhms)	Thickness (kHz)	Amplitude (kOhms)
1	386.0	8.9	5625.2	0.53
2	387.2	7.6	5591.6	0.49
3	390.8	8.4	5649.2	0.49
6	386.0	8.9	5614.4	0.49
7	390.8	7.5	5645.6	0.51
8	387.2	7.9	5630.0	0.58
9	386.0	8.5	5614.4	0.42
13	386.0	8.4	5588.0	0.50
14	383.6	7.8	5566.4	0.46
15	392.0	8.1	5636.0	0.45
Average	387.6	8.2	5616.1	0.49

**Table 2:** Pristine lap-shear coupon failure loads.

Pristine Coupon	Failure Load (N)
Batch 1—#1	10,984
Batch 1—#2	11,371
Batch 1—#3	9733
Batch 1—#4	11,137
Batch 2—#5	1423
Batch 2—#6	7804
Batch 3—#13	6213
Batch 3—#14	8874

**Table 3:** Embedded sensor coupon failure loads.

Embedded Sensor Coupon	Failure Load (N)
#7	3132
#8	7976
#15	7166
#16	7922

**Table 4:** Failure loads—second experiments.

Coupon	Failure Load (N)
1—Pristine	8326
2—Pristine	8496
3—Pristine	7781
4—Sensor	8093
5—Sensor	8221
6—Sensor	8554

## Data Availability

The data presented in this study are available upon request from the corresponding author.
